# Determination of pollutants, antibiotics, and drugs in surface water in Italy as required by the third EU Water Framework Directive Watch List: method development, validation, and assessment

**DOI:** 10.1007/s11356-024-32025-6

**Published:** 2024-01-27

**Authors:** Luisa Colzani, Carola Forni, Laura Clerici, Salvatore Barreca, Pierluisa Dellavedova

**Affiliations:** 1 ARPA Lombardia via Ippolito Rosellini n, 17 20124 Milan, Italy; 2 Department of Chemical Sciences, University of Catania, Viale A. Doria 6, 95100 Catania, Italy

**Keywords:** Watch List, Method developed and validation, LC-MS/MS

## Abstract

In this paper, we report a study concerning the quantification of new emerging pollutants in water as a request from the third European Watch List mechanism. The EU Watch List compound was investigated by an internal method that was validated in terms of detection limits, linearities, accuracy, and precision in accordance with quality assurance criteria, and it was used to monitor several rivers from 11 Italian regions. The methodology developed was satisfactorily validated from 5 to 500 ng L^−1^ for the emerging pollutants studied, and it was applied to different river waters sampled in Italy, revealing the presence of drugs and antibiotics. Rivers were monitored for 2 years by two different campaigns conducted in 2021 and 2022. A total of 19 emerging pollutants were investigated on 45 samples. The most detected analytes were O-desmethylvenlafaxine and venlafaxine. About azole compounds, sulfamethoxazole, fluconazole, and Miconazole were found. About antibiotics, ciprofloxacin and amoxicillin were found in three and one samples, respectively. Moreover, statistical analyses have found a significant correlation between O-desmethylvenlafaxine with venlafaxine, sulfamethoxazole with venlafaxine, and fluconazole with venlafaxine.

## Introduction

Analysis of emerging pollutants is an increasingly challenging aspect in the assessment of natural water quality so method development and water monitoring activities have attracted the scientific community's attention (Barreca et al. 2018; Barreca et al. 2020; Sun et al. 2022; Ren et al. 2023; Jiang and Dai 2023).

Moreover, the European Union (EU) has adopted several actions to prevent water contamination (Directive 2008/105/EC). In detail, by the Water Framework Directive (WFD), the EU has taken measures to tackle the pollution of freshwater ecosystems (Directive 2000/60/EC) through a system of structured prioritization.

To achieve the protection needed for surface water, a number of analytes are set out in the Water Framework Directive.

These are aimed at preventing or limiting the input of pollutants into water, preventing the deterioration in the status of water bodies, achieving good water status, and reversing any significant and sustained upward environmentally significant trends in pollutant concentrations.

To satisfy these requirements, a Watch List (WL) concerning emerging pollutants was published. Based on the mechanism introduced by Directive 2013/39/EU, the WL aims to better assess risks from chemicals found in surface water by monitoring data on potential water pollutants for which scarce monitoring data or data of insufficient quality are available.

The surface water Watch List (WL) under the Water Framework Directive (WFD) is a mechanism for obtaining high-quality union-wide monitoring data on potential water pollutants for the purpose of determining the risk they pose, and thus whether Environmental Quality Standards (EQS) is set for them at EU an level. Thus, the determination of pollutants in surface water has become an important topic in environmental science and water preservation, including analytical quantification at ultra-trace levels. For WL decision, specific or groups of emerging substances can be added during each update that occurs every 2 years. For these reasons, a Watch List mechanism was established to improve the available information on identifying the substances of the greatest concern.

The first WL, published by Decision (EU) 2015/495, included several substances, such as sunscreens, drugs, hormones, neonicotinoids, pesticides, and antibiotics (EU Decision 2015/495), and subsequent revisions have updated the list of substances to be monitored. In June 2018, the second updated version of the EU Watch List made its appearance (EU Decision 2018/840), and the third updated version of the EU Watch List was published in August 2020 (EU 2020/1161).

In the last 7 years, the member states of the EU have carried out monitoring actions to determine pollutants reported in the WL, and several data dissemination and analytical protocols were carried out by meeting. Some authors have reported the presence of hormones in surface waters both in Italy (Barreca et al. 2019) and in some areas of northern Europe (Simon et al. 2022), while other researchers have revealed the presence of antibiotics in hospital wastewater effluent collected from a hospital located in the southern zone of Madrid (Spain) and Catalonia (Spain) (Lopez et al. 2022; Gusmaroli et al. 2019).

In our previous work, a study concerning a method able to detect contaminant stability was validated for the analysis of WL contaminants in surface water (SW) (Barreca et al. 2021a), and the methodologies to improve contaminant stability in water were used to determine WL compounds in this study.

In the present work, we report one of the first water quality monitoring results in Europe about drug, pesticides, fluconazole compounds, and antibiotics. The study spans 2 years and focuses on the determination of 19 emerging contaminants in 45 samples from rivers located in Italy, in accordance with the Water Framework Directive (EU) 2020/1161. Whole water samples were extracted using a newly validated internal method based on the Horizon Solid Phase Extraction system, followed by determination using UHPLC-MS/MS

## Materials and methods

### Chemicals and reagents

Analytes used for qualitative and quantitative determinations were purchased from LabService Analytica. In detail, single solution at 100 μg L^−1^ of ciprofloxacin, trimethoprim, sulfamethoxazole, O-desmethylvenlafaxine, velanfaxine, fluconazole, miconazole, clotrimazole, ipconazole, imazalil, prochloraz, metconazole, tebuconazole, penconazole, tetraconazole, metaflumizone, dimoxystrobin, famoxadone, in acetonitrile (ANC) were used.

Amoxicillin solution at 100 μg L^−1^ in water was obtained by dissolving solid amoxicillin salt.

Isotopically labeled compounds (venlafaxine D-6, imidacloprid D-4, tetraconazole D-9, fluconazole D-4, and ciprofloxacin 13C) were purchased from LabService Analytica in ACN solution at 100 μg L^−1^. The purity grade of all standards used was always above 95%.

Methanol (MeOH) and acetonitrile (ACN) grades were bought from MERK, while water was obtained from the Milli-Q system.

Formic acid and ammonium formate were from Sigma-Aldrich.

### Sample extraction and clean-up

A total of 45 surface water samples were collected from different regions of Italy in accordance with EU sampling directives and shipped to the laboratory of the Regional Environmental Protection Agency of Lombardia (ARPA Lombardia).

For each year (2021–2022), two sampling campaigns were carried out respectively in April/May and July/August in order to investigate possible seasonal variations both for 2021 and 2022.

Water samples were collected in 1 L PP or glass bottles, covered by aluminum foil, and refrigerated at 4 °C during transport and storage.

Extractions were performed with an automatic Solid Phase Extraction (SPE) Horizon SPEDEX 5000 system using 47 mm diameter Empore™ SPE disks (active group polystyrene-divinylbenzene (SDB-XC) as sorbent phase. Elution processes were carried out involving elution of the sample, washing of the particulate collected on the filter with ACN, and reunification of the organic phase with aqueous eluate.

Extraction operating conditions are reported in Table 1, while extraction procedures are reported in detail in a previous paper (Barreca et al. 2021b). Briefly, an aliquot of 100 mL of sample was added to a 2.5 mL solution of labeled Standards and loaded on Horizon Spedex 5000 extraction system equipped with the SPE disk. The analyses are carried out both on non-acidified samples and acidified samples for amoxicillin determination.

**Table 1:** Conditions for SPE disk extraction procedures

Method steps	Eluent used	Exhaust line or sample line
Condition SPE disk	10 mL acetonitrile	Exhaust line
Load sample	100 mL samples	Sample line
Elute sample container	25 mL acetonitrile	Sample line
Air dry disk timer	30 s by nitrogen	Sample line
Pause	--	--
Clean system	20 mL methanol/water 50/50	Exhaust line

### LC-MS instrumentation

It used a liquid chromatographic system EXION LC SCIEX equipped with a binary pump EXION LC Sciex pump, a DGU-20A 5R degassing unit, a SIL-30AC containing a 50 μL loop, a CTO/20AC thermostat column compartment, and a CBM-20A module (Table 2).

**Table 2:** Chromatographic gradient mode

Time (min)	Solvent A (%)	Solvent B (%)
0	10	90
9	98	2
10	98	2
12	10	90

Chromatographic separation was performed on Restek CORTECS T3 analytical column (150 mm; 4.6 mm; 5 μm) using a mixture mobile phase of formic acid 0.05% + ammonium formate 5 mM in water (solvent A) and formic acid 0.05 % + ammonium formate 5mM in methanol (Solvent B) at a flow rate of 0.35 mL min^−1^ in gradient mode.

Analytes were detected by a 6500 plus Q-Trap mass spectrometer (Sciex), equipped with a Turbo V in-column interface by an Electrospray Ionization (ESI) probe operating in dual mode. Source and ion funnel parameters, as well as the precursor and product ions monitored, are reported in Tables 3 and 4.

**Table 3:** Electro spray ionization parameters

Parameter	Measure unit	Value
Curtain gas (CUR)	psi	35
Collision gas	-	Medium
Ion spray voltage (IS)	V	5500
Temperature TEM (GS2)	°C	400
Ion source gas (GS1)	psi	55
Ion source gas (GS2)	psi	60

**Table 4:** Analyte, m/z transitions, and operating parameters by ESI-MS in negative or positive mode. Quantification ion (Q1 as (m/z)), confirmation ion (Q3) as (m/z)), declustering potential (DP), entrance potential (EP), collision energy (CE), collision exit potential, (CXP), first transition (−1), second transition (−2)

Analyte	Q1	Q3	(DP)	(EP)	(CE)	(CXP)
Sulfamethoxazole-1	254	156	46	10	24	10.0
Sulfamethoxazole-2	254	92	46	10	36	9.5
Trimethoprim-1	291	230	40	10	30	10.0
Trimethoprim-2	291	123	40	10	30	10.0
Velanfaxine-1	278	260	20	10	15	10.0
Velanfaxine-2	278	121	20	10	35	10.0
O-desmethylvenlafaxine-1	264	246	40	10	20	10.0
O-desmethylvenlafaxine-2	264	107	40	10	60	9.5
Clotrimazole-1	277	242	20	10	25	9.5
Clotrimazole-2	277	163	20	10	70	9.5
Fluconazole-1	307	238	40	10	25	9.5
Fluconazole-2	307	220	40	10	22	9.5
Miconazole-1	417	161	40	10	25	9.5
Miconazole-2	417	159	40	10	25	9.5
Imazalil-1	297	159	81	10	25	9.5
Imazalil-2	297	201	81	10	25	9.5
Ipconazole-1	334	70	81	10	37	9.5
Ipconazole-2	334	125	101	10	48	9.5
Metconazole-1	320	70	100	10	50	5.0
Metconazole-2	320	125	90	10	35	5.0
Penconazole-1	284	70	81	10	37	8.0
Penconazole-2	284	159	81	10	35	15.0
Prochloraz-1	376	308	51	10	15	10.0
Prochloraz-2	376	70	51	10	43	5.0
Tetraconazole-1	372	159	86	10	35	8.0
Tetraconazole-2	372	70	86	10	48	12.0
Tebuconazole-1	308	70	86	10	51	8.0
Tebuconazole-2	308	125	86	10	55	6.0
Dimoxystrobin-1	327	116	66	10	29	6.0
Dimoxystrobin-2	327	205	66	10	23	14.0
Famoxadone-1	392	331	46	10	13	10.0
Famoxadone-2	392	238	46	10	23	15.0
Metaflumizone-1	507	178	70	10	35	10.0
Metaflumizone-2	507	116	70	10	30	10.0
Ciprofloxacin-1	332	231	50	10	48	10.0
Ciprofloxacin-2	332	314	50	10	31	10.0
Amoxicillin-1	366	208	25	10	16	10.0
Amoxicillin-2	366	114	25	10	16	10.0

Analytes were identified by comparison with related standards based on retention time matching and abundance ratios of multiple-reaction monitoring (MRM) transitions.

Quantitative analyses were carried out by the first ion mass transition (−1), and qualitative analyses were carried out by the second ion mass transition (−2).

### Calibration

Calibrations were performed by comparing different solutions obtained by serial dilution from intermediate solutions. In detail, 25 μL of the single standard at 100 μg mL^−1^ was diluted to 10 mL in ACN (solution A). Two hundred microliter solution A was diluted to 10 mL in ACN to obtain a mix solution containing 5 μg L^−1^ (solution B) of the single compound. Calibration standard solutions ranging from 5 to 500 ng L^−1^ were prepared by serial dilution from solution B in water and ACN mixture (75:25).

Calibration range for each analyte is reported in Table 5.

**Table 5:** Limit of quantification (LOQ) and calibration range for each analyte

Analyte	LOQ (ng L^−1^)	Calibration range (ng L^−1^)
Sulfamethoxazole	10	10–500
Trimethoprim	10	10**–**500
Velanfaxine	5	5**–**500
O-desmethylvenlafaxine	5	5**–**500
Clotrimazole	10	10**–**500
Fluconazole	10	10**–**500
Miconazole	10	10**–**500
Imazalil	10	10**–**500
Ipconazole	10	10**–**500
Metconazole	10	10**–**500
Penconazole	10	10**–**500
Prochloraz	10	10**–**500
Tetraconazole	10	10**–**500
Tebuconazole	10	10**–**500
Dimoxystrobin	10	10**–**500
Famoxadon	5	5**–**500
Metaflumizone	50	50**–**500
Ciprofloxacin	50	50**–**500
Amoxicillin	50	50**–**500

Intermediate mixed solutions containing all compounds were prepared weekly.

Water acetonitrile (75:25) working standard solutions were freshly prepared for every analytical batch analysis to avoid precipitations and degradation processes.

High-purity water was prepared using a Millipore Milli-Q purification system.

## Results

### Validation and quality assurance

By considering that for most of the investigated compound, it is very difficult to use a single standardized/official procedure, an internal method was developed. Prior to performing analyses, the method was validated by considering the principal issue for analytical chemistry analyses. Method validation was performed by considering both UNI EN ISO 17025 2018 and European SANTE 2016.

The analytical procedure was validated by analyzing spiked samples and by investigation of the following parameters:

- Selectivity was guaranteed by the use of specific SRM transitions and labeled standards, and it was tested by analyses on spiked matrices.
- Linearity was evaluated by making calibration, curves analyzing standard solutions prepared in a solvent mixture of Milli-Q water, and ACN (75.25) at different concentration levels as reported in Table 4. Linearity was evaluated good when the determination coefficient *R*^2^ was > 0.997. For all analytes, determination coefficients were from 0.997 to 0.999.
- Accuracy (expressed as percentage recovery) and precision (repeatability, expressed as relative standard deviation in percentage) were evaluated by analyzing spiked samples at a minimum of three different levels. For all analytes considered, accuracy ranged from 70 to 130% in good accordance with the validation performance reported in the guidelines.
- Limit of quantifications (LOQs) were determined as 10 times the standard deviation (S*r*) of signal at the first calibration level for each analyte. For all analytes investigated, obtained LOQs (see Table 6) were in good accordance with the European Union requirements.

**Table 6:** Validation results: accuracy and repeatability obtained at different levels

Analyte	Spiked concentration ng/L	Average (ng/L)	Recovery %	CV%
Sulfamethoxazole	50	46.74	93.48	9.88
	250	243.40	97.38	5.85
	500	549.00	109.8	8.39
Trimethoprim	50	45.01	90.02	4.91
	250	220.33	88.13	7.58
	500	511.3	102.3	7.72
Velanfaxine	5	4.99	99.87	4.29
	10	9.74	97.43	11.78
	50	47.54	95.08	4.95
	250	242.11	96.84	5.43
	500	517.17	103.43	5.59
O-desmethylvenlafaxine	5	5.79	115.74	5.55
	10	10.65	106.53	11.44
	50	50.58	101.16	9.70
	250	229.44	91.78	6.44
	500	584.50	109.70	5.71
Clotrimazole	10	8.36	83.60	9.64
	50	49.83	99.66	12.16
	250	245.11	98.04	9.10
	500	526.83	105.37	7.29
Fluconazole	50	48.783	97.45	10.22
	250	231.00	92.40	11.59
	500	547.50	109.50	10.76
Miconazole	50	40.04	80.08	5.25
	250	231.89	92.76	10.49
	500	483	96.60	9.96
Imazalil	50	50.837	101.68	8.95
	250	233.89	93.56	11.56
	500	465.15	93.03	8.41
Ipconazole	10	9.58	95.82	9.29
	50	55.14	110.29	10.65
	250	259.11	103.64	6.01
	500	581.67	116.33	10.27
Metconazole	10	9.57	95.70	11.26
	50	53.82	107.64	12.18
	250	258.44	103.38	7.31
	500	505.50	101.10	11.68
Penconazole	50	60.33	120.66	7.70
	250	270.89	108.36	5.57
	500	550.83	110.17	8.59
Prochloraz	50	52.57	105.14	8.34
	250	247.44	98.98	7.18
	500	502.50	100.50	7.47
Tetraconazole	50	57.94	115.88	5.17
	250	268.99	107.56	3.84
	500	579.50	115.90	5.15
Tebuconazole	50	58.03	116.06	5.79
	250	272.11	108.84	5.08
	500	565.83	113.17	3.36
Dimoxystrobin	10	9.01	90.1	9.71
	50	46.98	93.96	6.34
	250	236.44	94.58	7.01
	500	489.83	97.97	7.94
Famoxadone	5	5.45	108.92	8.70
	10	9.64	96.40	8.92
	50	43.39	86.78	9.64
	250	231.78	92.71	7.00
	500	541.67	108.33	9.96
Metaflumizone	50	53.62	107.25	8.95
	250	263.83	105.53	8.84
	500	479.83	95.97	10.22
Ciprofloxacin	50	46.32	92.63	8.25
	250	256.34	105.53	8.84
	500	475.56	95.11	5.32
Amoxicillin	100	82.87	82.87	6.66
	250	203.83	81.53	7.50
	500	448.73	89.75	6.09

Validation results are resumed and reported in Table 6.

Finally, uncertainties were calculated by mixed uncertainty quantification approach using Measurement Uncertainty Kit SW MUKIT (Nord-test 737). All calculated uncertainties were lower than 44% (uncertainty target), and the results are reported in Table 7.

**Table 7:** Extended uncertainty for each investigated analyte

Analyte	Uncertainty %
Trimethoprim	32
Venlafaxine	39
O-desmethylvenlafaxine	43
Clotrimazole	43
Fluconazole	40
Miconazole	43
Imazalil	44
Ipconazole	36
Metconazole	40
Penconazole	41
Prochloraz	30
Tetraconazole	34
Tebuconazole	34
Dimoxystrobin	35
Famoxadone	35
Metaflumizone	30
Amoxicillin	30
Ciprofloxacin	30
Sulfamethoxazole	36

In the context of quality assurance, blank solutions were analyzed prior to each analysis and every ten samples. The resulting target signals were examined, and acceptance criteria were applied, requiring values to be less than half of the limit of quantification (LOQ). Additionally, an independent standard solution at the middle level was employed to validate the calibration curve. To monitor instrumental drift, low and middle-level calibration solutions were run after every ten samples. The bias for quality control samples was in the range of ±20% for low and middle levels, respectively.

### River water analyses

**Fig. 1:**
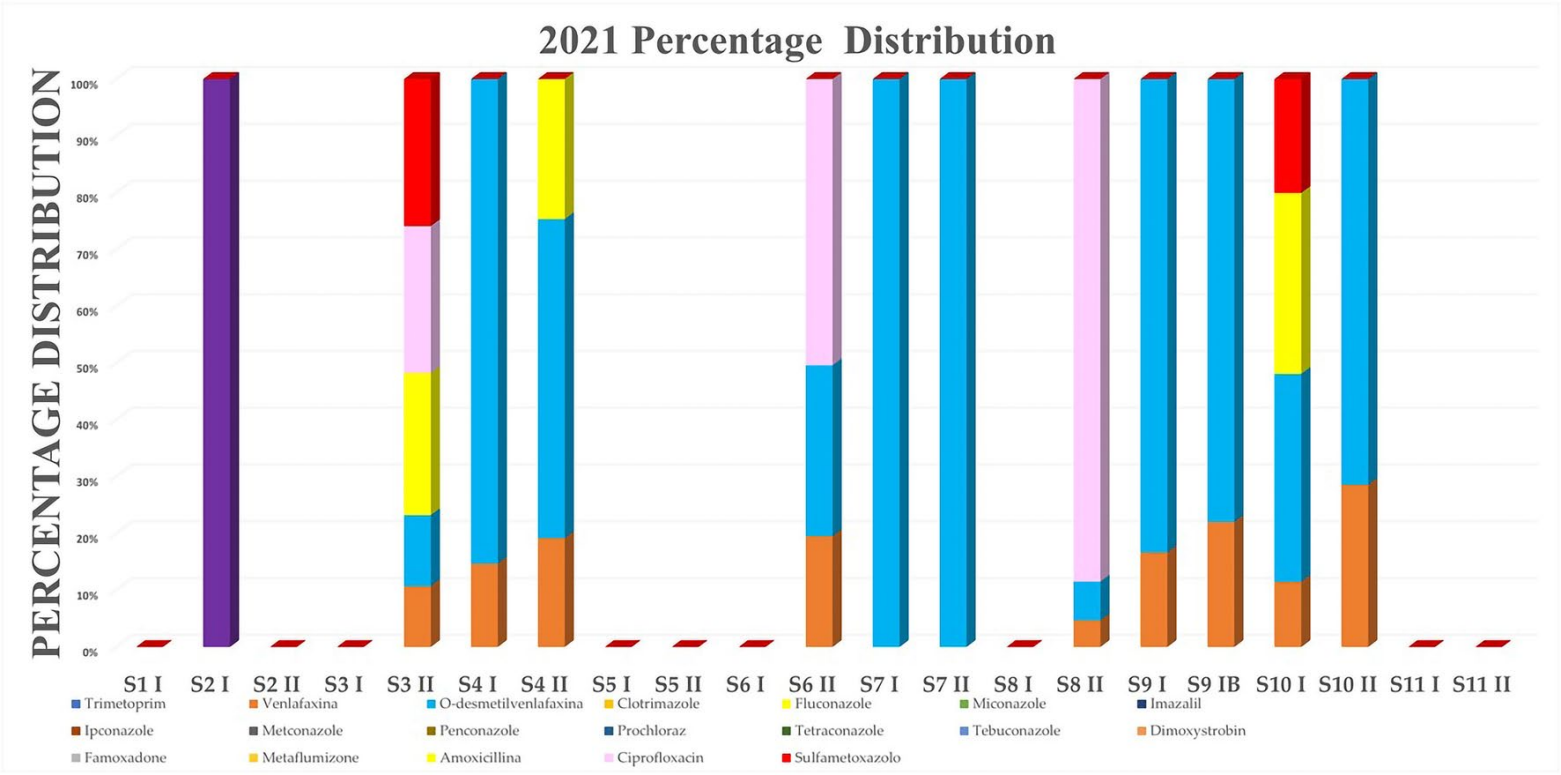
Analytes percentage distribution for 2021. Venlafaxine, O-desmethylvenlafaxine, fluconazole, miconazole, amoxicillin, ciprofloxacin, and sulfamethoxazole

**Fig. 2:**
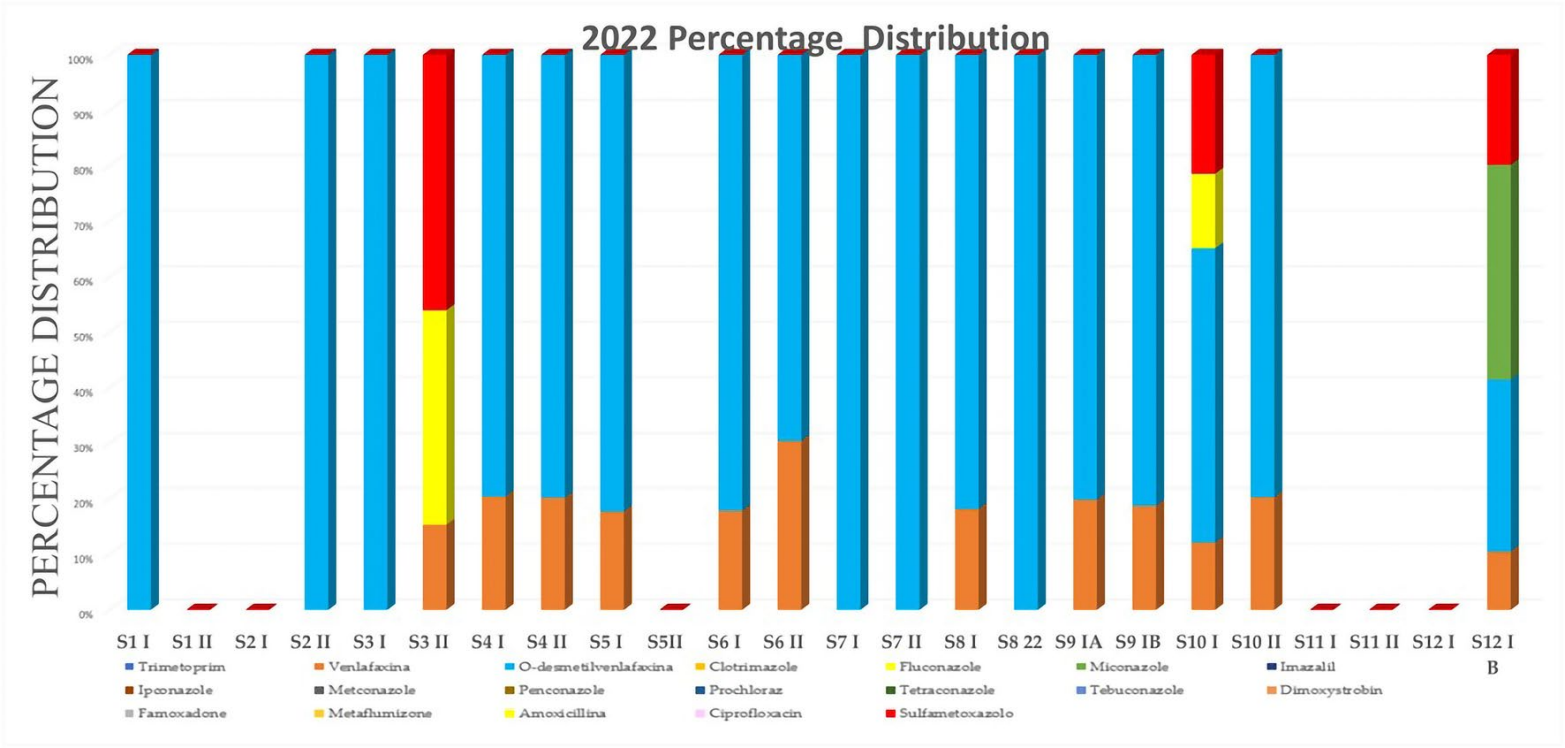
Analytes percentage distribution for 2022. Venlafaxine, O-desmethylvenlafaxine, fluconazole, miconazole, amoxicillin, ciprofloxacin, and sulfamethoxazole

**Fig. 3:**
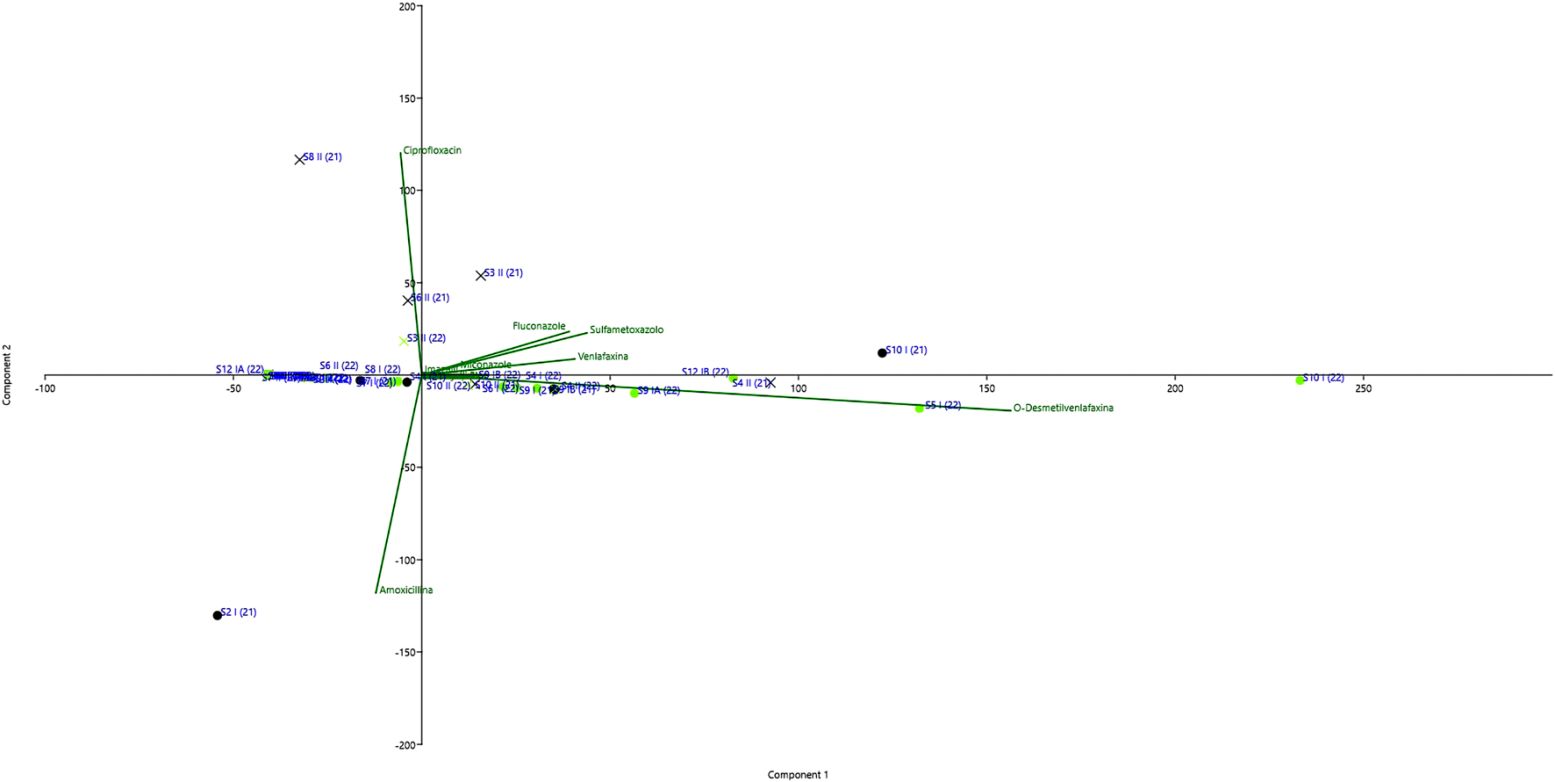
Samples of the second sampling of 2021

The analyte concentrations and percentage distributions for the samples collected during 2021–2022 years and analyzed by ARPA Lombardia laboratory are reported in Tables 8 and 9 and are shown in Figs. 1 and 2. Moreover, in order to better visualize the frequency of compounds, a radar chart is reported in Fig. 3. On the best of the us know, limited data are currently available concerning the third WL substances, and comparing results is a critical point of view.

**Table 8:** Results of pollutant determination on samples collected during 2021

	S1 I ng L^−1^	S2 I ng L^−1^	S2 II ng L^−1^	S3 I ng L^−1^	S3 II ng L^−1^	S4 I ng L^−1^	S4 II ng L^−1^	S5 I ng L^−1^	S5 II ng L^−1^	S6 I ng L^−1^	S6 II ng L^−1^	S7 I ng L^−1^	S7 II ng L^−1^	S8 I ng L^−1^	S8 II ng L^−1^	S9 I ng L^−1^	S9 IB ng L^−1^	S10 I ng L^−1^	S10 II ng L^−1^	S11 I ng L^−1^	S11 II ng L^−1^
Trimethoprim	< LOQ	< LOQ	< LOQ	< LOQ	< LOQ	< LOQ	< LOQ	< LOQ	< LOQ	< LOQ	< LOQ	< LOQ	< LOQ	< LOQ	< LOQ	< LOQ	< LOQ	< LOQ	< LOQ	< LOQ	< LOQ
Venlafaxine	< LOQ	< LOQ	< LOQ	< LOQ	24	6.7	42	< LOQ	< LOQ	< LOQ	24	< LOQ	< LOQ	< LOQ	9	16	22	39	22	< LOQ	< LOQ
O-desmethylvenlafaxine	< LOQ	< LOQ	< LOQ	< LOQ	28	39	123	< LOQ	< LOQ	< LOQ	37	27	9	< LOQ	13	80	78	124	55	< LOQ	< LOQ
Clotrimazole	< LOQ	< LOQ	< LOQ	< LOQ	< LOQ	< LOQ	< LOQ	< LOQ	< LOQ	< LOQ	< LOQ	< LOQ	< LOQ	< LOQ	< LOQ	< LOQ	< LOQ	< LOQ	< LOQ	< LOQ	< LOQ
Fluconazole	< LOQ	< LOQ	< LOQ	< LOQ	56	< LOQ	54	< LOQ	< LOQ	< LOQ	< LOQ	< LOQ	< LOQ	< LOQ	< LOQ	< LOQ	< LOQ	108	< LOQ	< LOQ	< LOQ
Miconazole	< LOQ	< LOQ	< LOQ	< LOQ	< LOQ	< LOQ	< LOQ	< LOQ	< LOQ	< LOQ	< LOQ	< LOQ	< LOQ	< LOQ	< LOQ	< LOQ	< LOQ	< LOQ	< LOQ	< LOQ	< LOQ
Imazalil	< LOQ	< LOQ	< LOQ	< LOQ	< LOQ	< LOQ	< LOQ	< LOQ	< LOQ	< LOQ	< LOQ	< LOQ	< LOQ	< LOQ	< LOQ	< LOQ	< LOQ	< LOQ	< LOQ	< LOQ	< LOQ
Ipconazole	< LOQ	< LOQ	< LOQ	< LOQ	< LOQ	< LOQ	< LOQ	< LOQ	< LOQ	< LOQ	< LOQ	< LOQ	< LOQ	< LOQ	< LOQ	< LOQ	< LOQ	< LOQ	< LOQ	< LOQ	< LOQ
Metconazole	< LOQ	< LOQ	< LOQ	< LOQ	< LOQ	< LOQ	< LOQ	< LOQ	< LOQ	< LOQ	< LOQ	< LOQ	< LOQ	< LOQ	< LOQ	< LOQ	< LOQ	< LOQ	< LOQ	< LOQ	< LOQ
Penconazole	< LOQ	< LOQ	< LOQ	< LOQ	< LOQ	< LOQ	< LOQ	< LOQ	< LOQ	< LOQ	< LOQ	< LOQ	< LOQ	< LOQ	< LOQ	< LOQ	< LOQ	< LOQ	< LOQ	< LOQ	< LOQ
Prochloraz	< LOQ	< LOQ	< LOQ	< LOQ	< LOQ	< LOQ	< LOQ	< LOQ	< LOQ	< LOQ	< LOQ	< LOQ	< LOQ	< LOQ	< LOQ	< LOQ	< LOQ	< LOQ	< LOQ	< LOQ	< LOQ
Tetraconazole	< LOQ	< LOQ	< LOQ	< LOQ	< LOQ	< LOQ	< LOQ	< LOQ	< LOQ	< LOQ	< LOQ	< LOQ	< LOQ	< LOQ	< LOQ	< LOQ	< LOQ	< LOQ	< LOQ	< LOQ	< LOQ
Tebuconazole	< LOQ	< LOQ	< LOQ	< LOQ	< LOQ	< LOQ	< LOQ	< LOQ	< LOQ	< LOQ	< LOQ	< LOQ	< LOQ	< LOQ	< LOQ	< LOQ	< LOQ	< LOQ	< LOQ	< LOQ	< LOQ
Dimoxystrobin	< LOQ	< LOQ	< LOQ	< LOQ	< LOQ	< LOQ	< LOQ	< LOQ	< LOQ	< LOQ	< LOQ	< LOQ	< LOQ	< LOQ	< LOQ	< LOQ	< LOQ	< LOQ	< LOQ	< LOQ	< LOQ
Famoxadone	< LOQ	< LOQ	< LOQ	< LOQ	< LOQ	< LOQ	< LOQ	< LOQ	< LOQ	< LOQ	< LOQ	< LOQ	< LOQ	< LOQ	< LOQ	< LOQ	< LOQ	< LOQ	< LOQ	< LOQ	< LOQ
Metaflumizone	< LOQ	< LOQ	< LOQ	< LOQ	< LOQ	< LOQ	< LOQ	< LOQ	< LOQ	< LOQ	< LOQ	< LOQ	< LOQ	< LOQ	< LOQ	< LOQ	< LOQ	< LOQ	< LOQ	< LOQ	< LOQ
Amoxicillin	< LOQ	191	< LOQ	< LOQ	< LOQ	< LOQ	< LOQ	< LOQ	< LOQ	< LOQ	< LOQ	< LOQ	< LOQ	< LOQ	< LOQ	< LOQ	< LOQ	< LOQ	< LOQ	< LOQ	< LOQ
Ciprofloxacin	< LOQ	< LOQ	< LOQ	< LOQ	58	< LOQ	< LOQ	< LOQ	< LOQ	< LOQ	62	< LOQ	< LOQ	< LOQ	169	< LOQ	< LOQ	< LOQ	< LOQ	< LOQ	< LOQ
Sulfamethoxazole	< LOQ	< LOQ	< LOQ	< LOQ	58	< LOQ	< LOQ	< LOQ	< LOQ	< LOQ	< LOQ	< LOQ	< LOQ	< LOQ	< LOQ	< LOQ	< LOQ	68	< LOQ	< LOQ	< LOQ

**Table 9:** Results of pollutant determination on samples collected during 2022

	S1 I ng L^−1^	S1 II ng L^−1^	S2 I ng L^−1^	S2 II ng L^−1^	S3 I ng L^−1^	S3 II ng L^−1^	S4 I ng L^−1^	S4 II ng L^−1^	S5 I ng L^−1^	S5II ng L^−1^	S6 I ng L^−1^	S6 II ng L^−1^	S7 I ng L^−1^	S7 II ng L^−1^	S8 I ng L^−1^	S8 22 ng L^−1^	S9 IA ng L^−1^	S9 IB ng L^-1^	S10 I ng L^-1^	S10 II ng L^-1^	S11 I ng L^-1^	S11 II ng L^-1^	S12 I A ng L^-1^	S12 I B ng L^-1^
Trimethoprim	< LOQ	< LOQ	< LOQ	< LOQ	< LOQ	< LOQ	< LOQ	< LOQ	< LOQ	< LOQ	< LOQ	< LOQ	< LOQ	< LOQ	< LOQ	< LOQ	< LOQ	< LOQ	< LOQ	< LOQ	< LOQ	< LOQ	< LOQ	< LOQ
Venlafaxine	< LOQ	< LOQ	< LOQ	< LOQ	< LOQ	23	19	20	39	< LOQ	15	7	< LOQ	< LOQ	8	< LOQ	25	15	56	14	< LOQ	< LOQ	< LOQ	34
O-desmethylvenlafaxine	13	< LOQ	< LOQ	18	11	< LOQ	74	79	181	< LOQ	69	16	36	12	36	11	101	65	245	55	< LOQ	< LOQ	< LOQ	100
Clotrimazole	< LOQ	< LOQ	< LOQ	< LOQ	< LOQ	< LOQ	< LOQ	< LOQ	< LOQ	< LOQ	< LOQ	< LOQ	< LOQ	< LOQ	< LOQ	< LOQ	< LOQ	< LOQ	< LOQ	< LOQ	< LOQ	< LOQ	< LOQ	< LOQ
Fluconazole	< LOQ	< LOQ	< LOQ	< LOQ	< LOQ	58	< LOQ	< LOQ	< LOQ	< LOQ	< LOQ	< LOQ	< LOQ	< LOQ	< LOQ	< LOQ	< LOQ	< LOQ	62	< LOQ	< LOQ	< LOQ	< LOQ	< LOQ
Miconazole	< LOQ	< LOQ	< LOQ	< LOQ	< LOQ	< LOQ	< LOQ	< LOQ	< LOQ	< LOQ	< LOQ	< LOQ	< LOQ	< LOQ	< LOQ	< LOQ	< LOQ	< LOQ	< LOQ	< LOQ	< LOQ	< LOQ	< LOQ	125
Imazalil	< LOQ	< LOQ	< LOQ	< LOQ	< LOQ	< LOQ	< LOQ	< LOQ	< LOQ	< LOQ	< LOQ	< LOQ	< LOQ	< LOQ	< LOQ	< LOQ	< LOQ	< LOQ	< LOQ	< LOQ	< LOQ	< LOQ	< LOQ	< LOQ
Ipconazole	< LOQ	< LOQ	< LOQ	< LOQ	< LOQ	< LOQ	< LOQ	< LOQ	< LOQ	< LOQ	< LOQ	< LOQ	< LOQ	< LOQ	< LOQ	< LOQ	< LOQ	< LOQ	< LOQ	< LOQ	< LOQ	< LOQ	< LOQ	< LOQ
Metconazole	< LOQ	< LOQ	< LOQ	< LOQ	< LOQ	< LOQ	< LOQ	< LOQ	< LOQ	< LOQ	< LOQ	< LOQ	< LOQ	< LOQ	< LOQ	< LOQ	< LOQ	< LOQ	< LOQ	< LOQ	< LOQ	< LOQ	< LOQ	< LOQ
Penconazole	< LOQ	< LOQ	< LOQ	< LOQ	< LOQ	< LOQ	< LOQ	< LOQ	< LOQ	< LOQ	< LOQ	< LOQ	< LOQ	< LOQ	< LOQ	< LOQ	< LOQ	< LOQ	< LOQ	< LOQ	< LOQ	< LOQ	< LOQ	< LOQ
Prochloraz	< LOQ	< LOQ	< LOQ	< LOQ	< LOQ	< LOQ	< LOQ	< LOQ	< LOQ	< LOQ	< LOQ	< LOQ	< LOQ	< LOQ	< LOQ	< LOQ	< LOQ	< LOQ	< LOQ	< LOQ	< LOQ	< LOQ	< LOQ	< LOQ
Tetraconazole	< LOQ	< LOQ	< LOQ	< LOQ	< LOQ	< LOQ	< LOQ	< LOQ	< LOQ	< LOQ	< LOQ	< LOQ	< LOQ	< LOQ	< LOQ	< LOQ	< LOQ	< LOQ	< LOQ	< LOQ	< LOQ	< LOQ	< LOQ	< LOQ
Tebuconazole	< LOQ	< LOQ	< LOQ	< LOQ	< LOQ	< LOQ	< LOQ	< LOQ	< LOQ	< LOQ	< LOQ	< LOQ	< LOQ	< LOQ	< LOQ	< LOQ	< LOQ	< LOQ	< LOQ	< LOQ	< LOQ	< LOQ	< LOQ	< LOQ
Dimoxystrobin	< LOQ	< LOQ	< LOQ	< LOQ	< LOQ	< LOQ	< LOQ	< LOQ	< LOQ	< LOQ	< LOQ	< LOQ	< LOQ	< LOQ	< LOQ	< LOQ	< LOQ	< LOQ	< LOQ	< LOQ	< LOQ	< LOQ	< LOQ	< LOQ
Famoxadone	< LOQ	< LOQ	< LOQ	< LOQ	< LOQ	< LOQ	< LOQ	< LOQ	< LOQ	< LOQ	< LOQ	< LOQ	< LOQ	< LOQ	< LOQ	< LOQ	< LOQ	< LOQ	< LOQ	< LOQ	< LOQ	< LOQ	< LOQ	< LOQ
Metaflumizone	< LOQ	< LOQ	< LOQ	< LOQ	< LOQ	< LOQ	< LOQ	< LOQ	< LOQ	< LOQ	< LOQ	< LOQ	< LOQ	< LOQ	< LOQ	< LOQ	< LOQ	< LOQ	< LOQ	< LOQ	< LOQ	< LOQ	< LOQ	< LOQ
Amoxicillin	< LOQ	< LOQ	< LOQ	< LOQ	< LOQ	< LOQ	< LOQ	< LOQ	< LOQ	< LOQ	< LOQ	< LOQ	< LOQ	< LOQ	< LOQ	< LOQ	< LOQ	< LOQ	< LOQ	< LOQ	< LOQ	< LOQ	< LOQ	< LOQ
Ciprofloxacin	< LOQ	< LOQ	< LOQ	< LOQ	< LOQ	< LOQ	< LOQ	< LOQ	< LOQ	< LOQ	< LOQ	< LOQ	< LOQ	< LOQ	< LOQ	< LOQ	< LOQ	< LOQ	< LOQ	< LOQ	< LOQ	< LOQ	< LOQ	< LOQ
Sulfamethoxazole	< LOQ	< LOQ	< LOQ	< LOQ	< LOQ	69	< LOQ	< LOQ	< LOQ	< LOQ	< LOQ	< LOQ	< LOQ	< LOQ	< LOQ	< LOQ	< LOQ	< LOQ	99	< LOQ	< LOQ	< LOQ	< LOQ	64

In 57% of the analyzed samples in 2021 and 75% of the samples in 2022, it was found at least a contaminant. In both years, the most abundant pollutant was venlafaxine and its metabolite (O-desmethylvenlafaxine).

In detail, during analyses performed in 2021, O-desmethylvenlafaxine and venlafaxine were found in 52% and 43% of analyzed samples, fluconazole and ciprofloxacin in the 14%, sulfamethoxazole in the 10%, and amoxicillin was quantified in only one sample (5% of analyzed samples) (see Fig. 3).

About analyses performed in 2022, O-desmethylvenlafaxine and venlafaxine were found in 71% and 50% of the samples, sulfamethoxazole in 13%, while fluconazole and miconazole were quantified in 8% and 4% of samples, respectively.

The most contaminated river was the S 10 sample containing O-desmethylvenlafaxine, sulfamethoxazole, fluconazole, and venlafaxine. This trend which can be explained considering that S 10 river collects the outflows of wastewater treatment plant from an area with high anthropic impact. Furthermore, the highest concentrations of pollutants were found during the first sampling period.

In detail, in the first sampling conducted in 2021, O-desmethylvenlafaxine, fluconazole, sulfamethoxazole, and venlafaxine were quantified as 124, 108, 68, and 39 ng L^−1^, while only O-desmethylvenlafaxine and venlafaxine were found in the analyses performed in the second sampling at of 55 ng L^−1^ and 22 ng L^−1^ respectively.

Similar analytes and concentrations were detected in the samples collected in the first sampling in 2022 whit O-desmethylvenlafaxine and venlafaxine quantified as 245 and 56 ng L^−1^, sulfamethoxazole was quantified as 99 ng L^−1^, and fluconazole was quantified as 62 ng L^−1^. Moreover, as observed in 2021, a decrease in pollutants was detected in the samples collected during the second sampling investigation (O-desmethylvenlafaxine 55 ng L^−1^ and venlafaxine 14 ng L^−1^).

Venlafaxine is one of the most widely prescribed antidepressant drugs, and several studies detected venlafaxine in wastewater and surface water collected in the European Union (Schluesener et al. 2015) and the USA (Writer et al. 2013) together with its metabolite O-desmethylvenlafaxine.

M.P. Schlüsener et al. in 2012 detected O-desmethylvenlafaxine and venlafaxine in the Rhine River in Germany at 56 ng L^−1^ and 26 ng L^−1^, respectively, while the Minnesota Pollution Control Agency (MPCA) estimates that approximately 5% of stream miles in Minnesota have detectable levels of venlafaxine. In a recent study about the impacts of wastewater treatment plants (WWTPs) on the aquatic environment (Figuière et al. 2022), researchers have assessed that venlafaxine could have possible toxic effects on the environment.

About azole compounds, in 2021, sulfamethoxazole was found in two samples and fluconazole in three samples, while for 2022, sulfamethoxazole was found in three samples, fluconazole in two samples while miconazole in only one sample.

Sulfamethoxazole is an antibiotic used for bacterial infections such as urinary tract infections, bronchitis, and prostatitis, and it is effective against both gram-negative and positive bacteria.

It was introduced to the USA in 1961 and is now mostly used in combination with trimethoprim as recommended in the WHO Model List of Essential Medicines as a first-choice treatment for urinary tract infections (Roth et al. 2018).

The presence of other azole compounds in surface water can be explained by considering that fluconazole is an antifungal medicine. It is used to treat infections caused by different kinds of fungus and the most commonly used to treat many infections caused by virus. Moreover, as reported, fluconazole due to its relatively low lipophilicity and limited degree of binding to plasma proteins is only partially metabolized, so fluconazole concentrations are 10–20–fold higher in the urine than blood (Wildfeuer et al. 1997).

Miconazole belongs to a class of antifungal medications called imidazoles, and generally, it is used to treat fungal skin infection that causes a red scaly rash on different parts of the body (Nenoff et al. 2017). These substances can be considered widely consumed substances and as reported in literature, are often detected in municipal wastewater and surface waters globally (Grobin et al. 2022; Spurgeon et al. 2022).

In general, pharmaceutical drugs and byproducts were the most abundant groups in river water. This pattern is clearly visible in Figs. 1 and 2.

### Principal component analysis (PCA)

In order to underline possible correlations between samples or from samples to analytes or sampling campaign, authors have provided the use of multivariate data analysis called Principal Component Analysis (PCA). PCA is a common statistical technique to reduce variable numbers. It is a mathematical procedure that transforms a set of possibly correlated variables into a new set of uncorrelated variables called principal components. The main purpose of PCA is to maximize the amount of variance in the original dataset by projecting it onto a lower dimensional space while minimizing the loss of information.

To conduct PCA analysis, data were normalized by centering at the mean value.

In the present study, PCA analysis was carried out on concentrations of 19 pollutants in 45 samples.

A cumulative variance of 69.36 was explained by two eigenvectors–principal components. The first principal component (PC1) can explain 55.585% of the total variance, while the second (PC2) is 13.781%.

In Fig. 3, it is reported that PCA analysis results and data point were divided as

- Point in black refers to data concerning the first sampling campaign conducted during 2021.
- Cross in black refers to data concerning the second sampling campaign conducted during 2021.
- Point in red refers to data concerning the first sampling campaign conducted during 2022.
- Cross in red refers to data concerning the second sampling campaign conducted during 2022.

As shown in Fig. 3, only samples of the second sampling of 2021 were characterized by ciprofloxacin while only samples can be discriminated by amoxicillin, in good accordance with data reported in Tables 8 and 9 and underline the good predictive correlation od PCA1 and PCA2.

Moreover, a statistical analysis was performed on data obtained from PCA analyses data and, interesting correlations were found between several compounds.

**Fig. 4:**
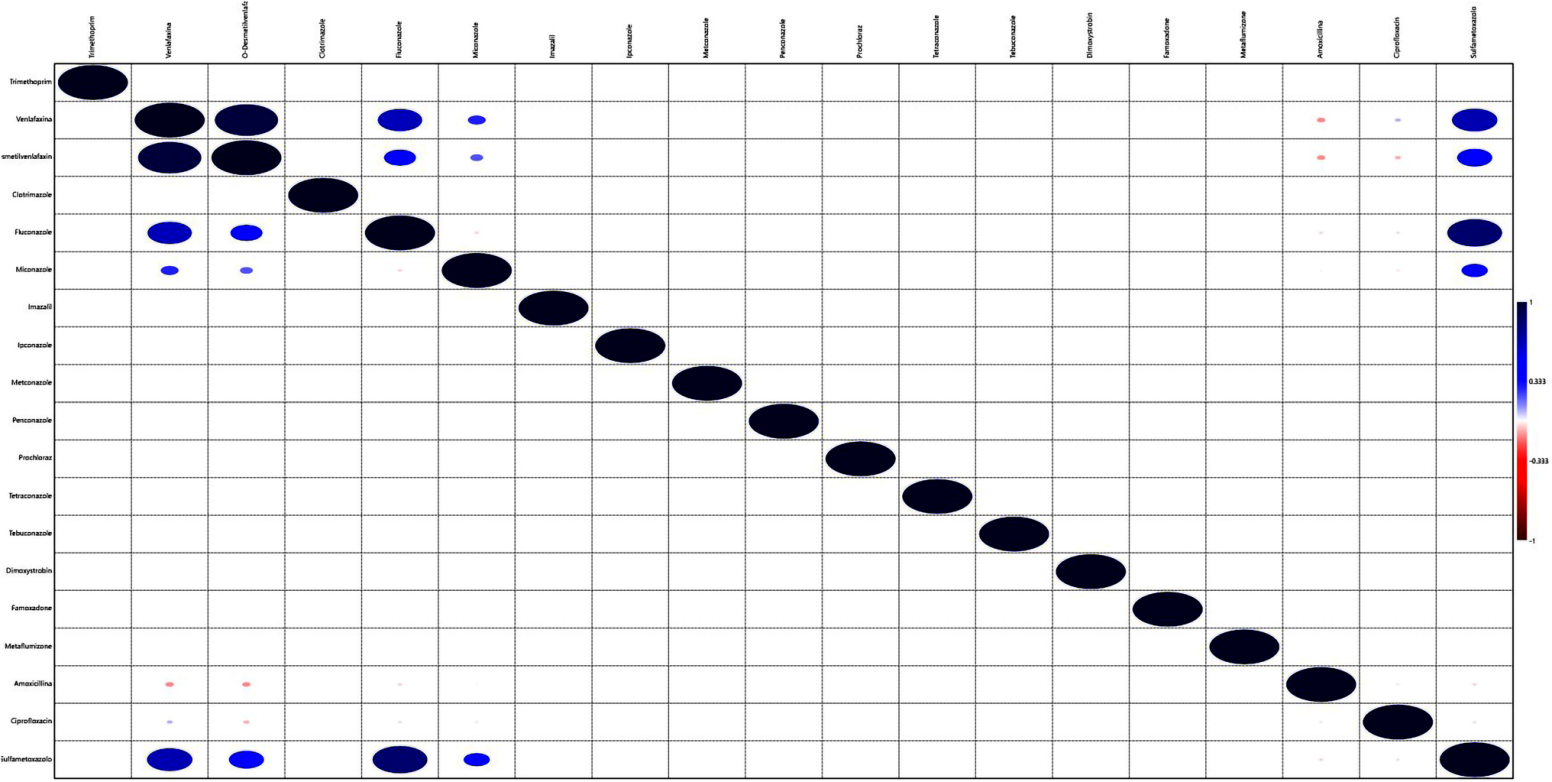
Significant correlations found between O-desmethylvenlafaxine with venlafaxine, sulfamethoxazole with venlafaxine, and fluconazole with venlafaxine

Indeed, as reported in Fig. 4, significant correlations were found between O-desmethylvenlafaxine with venlafaxine (*r* = 0.90), sulfamethoxazole with venlafaxine (*r* = 0.65), and fluconazole with venlafaxine (*r* = 0.63).

By considering that these drugs can be used for human applications, it is possible to hypothesize a common origin source.

## Conclusions

In this research paper, the analytical challenges associated with the determination of new emerging pollutants in water, in light of the requirements of the European Watch List, have been addressed. A monitoring of the 19 pollutants included in the third Watch List 2020/1161 was carried out in Italy rivers. Result shows that among the researched WL substances, fluconazole, sulfamethoxazole, venlafaxine and its metabolite O-desmethylvenlafaxine were the most detected.

The group of pharmaceuticals were the most abundant pollutants detected and, moreover, these substances were detected and quantified both in 2021 and 2022 analyses and are also classified as widely consumed substances and can be released into surface and ground waters from WWTP. For these reasons, it is preferred to keep monitoring the concentrations of these analytes, especially for venlafaxine and its metabolites, in surface and ground waters to ensure that their concentrations are not increasing.

## Data Availability

All data generated or analyzed during this study are with the authors, and, if necessary, she is available for taking any question about the datasets, and these can be requested by reasonable request.
